# Sequential Leaching and Mineralogical Controls of Rare Earth Elements and Yttrium Occurrence in Bituminous Coal from Upper Silesian Coal Basin (Poland)

**DOI:** 10.3390/ma19061066

**Published:** 2026-03-11

**Authors:** Zdzisław Adamczyk, Joanna Komorek

**Affiliations:** Faculty of Mining, Safety Engineering and Industrial Automation, Silesian University of Technology, ul. Akademicka 2, 44-100 Gliwice, Poland; joanna.komorek@polsl.pl

**Keywords:** rare earth elements (REE), bituminous coal, sequential chemical leaching, extractive metallurgy, mineral processing, mineral properties

## Abstract

In this study, the occurrence and leachability of rare earth elements and yttrium (REY) in medium-rank coal—meta-bituminous B coal from the southwestern part of the Upper Silesian Coal Basin in Poland—were investigated. The coal samples contained variable amounts of siderite, dolomite, calcite, kaolinite, illite, quartz, apatite, and pyrite in their mineral composition. A five-step sequential chemical leaching procedure was used, including deionized water, 3% HCl, 5% HNO_3_, 10% HNO_3_ with microwave assistance, and concentrated HCl–HF also with microwave assistance. The highest concentrations of ∑REY were observed in seam 404/1. Light REY (LREY) dominated the REY composition (>75%), while heavy REY (HREY) accounted for less than 10%. The chondrite-normalised REY patterns and total REY content indicate a clastic origin of REY-bearing minerals. The most efficient leaching occurred in stages IV and V. The solutions from stages I–III preferentially mobilised critical REY, while those from stages IV–V reflected the REY distribution in the coal. Based on the C_outl_ index, both coal and leachates from the later stages are classified as prospective REY resources. However, absolute REY concentrations should be considered when interpreting C_outl_ values. The positive correlation between apatite and kaolinite contents and ∑REE concentrations suggests their role in REY enrichment.

## 1. Introduction

The well-established role of rare earth elements and yttrium (REY) as essential to the development of modern technology is no longer disputed. Their use in the high-tech industry and in many products (computers, mobile phones, permanent magnets, medical and military equipment, etc.) has made them part of everyday use [1,2,3].

The high and increasing demand for REY, with relatively low production of REY (availability of deposits, resource constraints), creates a market supply risk [4,5,6]. While scenarios for the supply of neodymium, for example, have previously projected demand increases of 700% and 60% by 2037 and 2050, respectively [7,8], there is no doubt that this increase is inevitable.

The effect of the CO_2_ emission reductions set out in the EU Regulation [9] will result in the sale of new zero-emission cars from 2035. So, it is to be expected that battery-powered vehicles will be preferred to the currently more expensive alternatives (e.g., hydrogen fuel). However, the manufacture of electric cars, like that of wind turbines, requires the use of the most powerful and durable neodymium or samarium magnets in the motors, often doped with heavy rare earth elements (HREY—e.g., terbium and dysprosium), which increase the magnet’s thermal resistance [10,11].

The European Union has classified rare earth elements (REEs) into two groups (heavy and light REEs) out of thirty-four critical raw materials due to their role in the development of modern technologies, rapidly growing demand and supply risks, given the limited own deposits of these raw materials (EU Regulation [12]). While the European Raw Materials Alliance (ERMA) has identified projects in many Union countries to support the mining, separation, metallurgy, recycling, and production of REEs, these would, if fully implemented, meet, as ERMA estimates, only around 20% of the European demand [13].

For this reason, research has been ongoing for many years to identify alternative sources of these elements, including secondary sources [4,6,14,15,16,17,18]. The use of secondary sources can largely reduce the adverse environmental impacts of REY production. In this case, the impacts associated with the mining and/or enrichment of REY ores would be avoided; on the other hand, the form of occurrence in some secondary sources may allow for easier REY extraction, which can also largely reduce the adverse environmental impacts of REY production.

One of the alternative REY sources considered is power plant fly ash, which is generated as a result of the combustion of hard coal (CFA) and lignite (CFB), where there is a significant concentration of these metals as compared to the initial fuel [19,20,21]. The recovery of REY from CFA by acid leaching, sometimes with alkaline activation, is troublesome, as these elements during the solid fuel combustion process are incorporated into the dominant glassy aluminosilicate phase, which makes their dissolution difficult and requires strongly acidic conditions [22,23].

Power plants in Poland are mainly fired with hard coal and lignite. For this reason, a lot of research work is being carried out to assess the REY potential in the resulting fly ash and to seek to recover these metals [24,25,26,27,28].

However, to date, the results of studies on the content of REY in coal, their concentration in individual coal minerals, and the possibility of extracting REY directly from coal from Polish coalfields appear in only a few papers [29,30,31,32,33]. Mineral constituents known to accompany coal seams in the Upper Silesian Coal Basin in Poland (USCB), like in other deposits of this type in the world, include mainly silicates and aluminosilicates, carbonates, apatite, sulphides, and others.

Poland’s primary energy fuel has so far been coal, which, in an era of the development of clean coal technologies (CCT), renewable energy sources (RES), and restrictions on fuel imports from the Russian Federation, will be an important energy resource in the coming years [34]. At the same time, coal, as well as the by-products of its combustion, can provide an alternative source of REY in the future, which are essential for the development of RES.

The aim of the present study was to explore the distribution of rare earth elements and yttrium (REY) in the sequential chemical leaching of coal samples from strata seams in the south-western part of the Upper Silesian Coal Basin (USCB). For the purposes of this study, a modified five-step leaching procedure was applied, as described by D.A. Spears [35], which enables the identification of minerals in the coal attributable to REY concentration. To date, such detailed REY research in Polish coal has not been conducted. The presented results should be considered representative of the investigated seams within the studied area and not necessarily of the entire Upper Silesian Coal Basin.

## 2. Materials and Methods

The study was conducted on eight-channel samples of coal from seams in the south-western part of the Upper Silesian Coal Basin (USCB). Samples were taken in accordance with PN-ISO 13909 [36].

Samples were reduced, averaged, and ground to less than 1 mm, and grain specimens—blocks according to PN-ISO 7404-2 [37]—were prepared for microscopic examination. To determine the chemical and mineral composition, the samples were ground to less than 0.2 and 0.1 mm, respectively.

Measurements of the mean reflectance R of vitrinite and maceral group analysis (according to PN-ISO 7404-3 [38]) were carried out on a Axioskop microscope (Carl Zeiss, Jena, Germany) in reflected light with a microphotometer under the following conditions: immersion fluid with a refractive index n_o_ = 1.5176, temperature 23 °C, wavelength λ = 546 nm.

To determine the mineral composition, the ground samples were dressed in a mixture of tetrachloroethylene and toluene with a density of d = 1.40 g/cm^3^. The fractions with a density of d > 1.40 g/cm^3^ were subjected to X-ray phase analysis using an Empyrean diffractometer (PANalytical, Almelo, The Netherlands) under the following conditions: CuK_α_ radiation, range 2θ = 5–70°, step 0.02°, time 2 s. The quantitative composition was determined using the Rietveld refinement method with the HighScore plus v. 4.9 software from Malvern Panalytical.

The process of sequential chemical leaching of samples was performed using a five-step modified procedure described by D.A. Spears [35]. The applied sequential leaching procedure should be regarded as operational rather than strictly mineral-specific, and the obtained fractions represent approximate associations with geochemical hosts.

In step I, 15 mL of deionised water was added to a sample of approximately 0.5 g and shaken for 12 h. The suspension was centrifuged for 10 min at 4000× *g*. The solution was decanted into a volumetric flask and made up to 25 mL with deionised water, and the elements were quantified. The solution from step I contained elements bound in liquids contained in the coal pores (mainly hygroscopic water) and in water-soluble minerals (mainly sulphates).

In step II, 15 mL of diluted (3%) HCl was added to the solid residue from step I and shaken for 12 h. The suspension obtained was centrifuged for 10 min at 4000× *g*. The solution was decanted into a volumetric flask and made up to 25 mL with deionised water, and the elements were quantified. The solution obtained in step II contained elements associated with carbonate minerals (mainly calcite), monosulphides, and exchangeable cations.

In step III, 15 mL of diluted (5%) HNO_3_ was added to the solid residue from step II and subjected to extraction in a water bath at 80 °C for 30 min. The suspension was centrifuged in the same manner as in steps I and II, and the solution for elemental determinations was obtained after decanting and diluting with deionised water to 25 mL. The solution obtained in step III contained elements associated with pyrite and carbonate minerals (dolomite and ankerite).

In step IV, 10 mL of concentrated HNO_3_ was added to the solid residue from step III and subjected to microwave radiation-assisted digestion in a closed circuit in PTFE vessels. The resulting solution was transferred back to the flask and made up to 30 mL with concentrated HNO_3_ and then centrifuged for 10 min at 4000× *g*. The solution from this step was decanted and made up to 50 mL with deionised water. The elements quantified in it were associated with organic matter.

In step V, the solid residue from step IV was transferred to a PTFE vessel and, after the addition of 7.5 mL of concentrated HCl and 2.5 mL of HF, was subjected to microwave mineralisation. The final solution was evaporated to dryness, then the solid residue was dissolved in 10 mL of concentrated HNO_3_ and made up to 50 mL with deionised water. Elements associated with silicates were quantified in the resulting solution.

Compared to the original Spears [35] procedure, minor modifications were introduced, including changes in extraction conditions (use of a water bath instead of a hot plate), reagent volumes, and the proportions of the digestion mixture in the final step. These modifications do not alter the overall concept of the method but provide a more precise definition of the experimental conditions.

Sequential chemical leaching of elements concentrated in solutions from each step allowed the total REY amounts from steps I–III to be interpreted as elements associated with carbonate minerals and apatite, while those from steps IV–V were associated with organic matter and silicates. However, it should be noted that this interpretation is operational in nature and represents an approximate assignment to dominant geochemical hosts.

The REY content of the solutions obtained from each sequential chemical leaching step was determined by ICP-MS using a Varian 810-MS ICP mass spectrometer (Varian, Mulgrave, Australia) under the following conditions: RF power 1.4 kW, plasma flow rate 17 L/min, nebuliser flow rate 1.00 L/min. The following were used to plot the calibration curve: (i) solutions with concentrations in the range of 0.02 to 20 μg/L; (ii) P/N 4400-ICPMS1 multi-element standards, ICP-MS Concentration Verification Check Standard I (REY, 10 mg/L, Peak Performance CPI International, Santa Rosa, CA, USA) and single-element standard solutions (other elements, 1000 mg/L, Peak Performance, CPI International, Santa Rosa, CA, USA); and (iii) ultra-pure water (Simplicity Water Purification Systems, Merck Millipore, Burlington, VT, USA).

## 3. Results

### 3.1. Petrographic Composition of Coal

Vitrinite from the coal samples tested had a mean reflectance ranging from R_r_ = 1.10% (sample s4, seam 404/1 and s2, seam 403/1) to R_r_ = 1.16% (sample s5, seam 404/2) with a standard deviation in the range of s = (0.04–0.05%) (Table 1). This indicates a similar degree of coalification (rank) of the samples. According to the PN-ISO 11760:2007 classification [39], these samples represented medium rank coal—meta-bituminous B.

**Figure 1:**
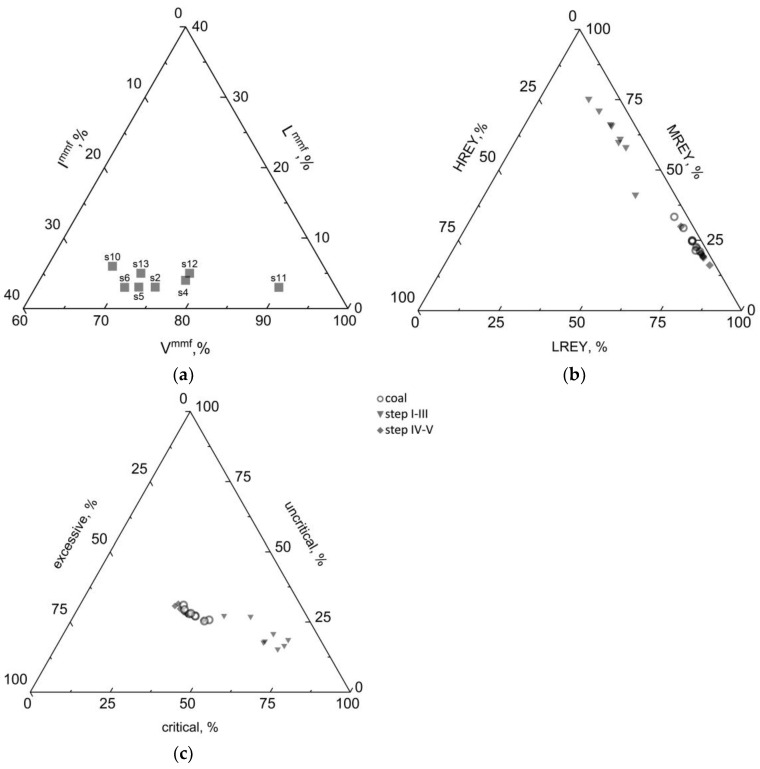
Shares of (**a**) maceral groups in the tested coal samples, (**b**) HREY, MREY, LREY, (**c**) critical, non-critical, and excess elements in the coal samples and in solutions obtained from their sequential chemical leaching. The classification of REY into critical, non-critical, and excessive groups follows the approach described in [4].

Analysis of the maceral groups revealed that the vitrinite content (V^mmf^) was usually greater than 60%, and in sample s11 it was as high as 90% (Table 1). Therefore, the coal samples tested were classified as moderately-high-vitrinite (60% ≤ V^mmf^ < 80%) and high-vitrinite (V^mmf^ > 80%) coals (PN-ISO 11760:2007). The content of liptinite in the coal samples tested (L^mmf^) varied within the range of L^mmf^ = (3–6%) (Table 1), while the proportion of inertinite varied over a wide range of I^mmf^ = (7–25%) (Table 1 and Figure 1a).

### 3.2. Mineral Matter in Coal

The mineral matter content of the samples varied from 1% to 14%, but only in samples s2 and s6 was it greater than 10%, whereas in the others it was no greater than 3% (Table 1). The mineral matter was represented by carbonate minerals and polymineral constituents made up of carbonates, clay minerals, and other very fine minerals difficult to identify by microscopy. Carbonate minerals occurred most frequently as cellular space fillers in semifusinite, less frequently as individual grains. The polymineral constituents were present as single grains and showed weak fluorescence in colours ranging from yellow to orange, probably related to the presence of bituminous matter in addition to the minerals. Clay minerals and iron sulphides were less common in the samples.

The presence of carbonate minerals (siderite, dolomite, occasional calcite), clay minerals (kaolinite, occasional illite), as well as quartz, pyrite, and phosphate minerals (apatite) was confirmed in the coal samples by XRD (X-ray diffraction).

The proportion of individual minerals in the coal samples varied, even in samples taken from the same seam (Table 2). And although kaolinite usually dominated (55.7–81.8%), in the samples from seam 404/2 its proportion was much lower (14.8–23.9%) to the benefit of dolomite (s5—47.4%) and siderite (s6—61.7%). What is noteworthy is the much lower proportion of total carbonates in the samples from the other coal seams (1.4–20.0%); in addition, dolomite was found in five out of eight samples tested. The quantitative contribution of calcite was only marginal. Apatite (5.3–29.3%) and quartz (6.4–23.2%) were also quantitatively significant minerals, the presence of which, like dolomite, was not found in all samples. Illite, found in only one sample (s4), became significant in this sample due to its quantity (10.4%), as compared to quartz (6.4%) and siderite (1.4%). Pyrite was only present in seam 403/1 (s11 and s12). From the point of the distribution of REY in the samples studied, apatite may be of greatest importance due to its role in their concentration. However, the role of those minerals that are found in the greatest quantities in individual samples—clay minerals and carbonates—cannot be underestimated.

### 3.3. Concentration of REY in Sequential Coal Leaching Solutions

**Figure 2:**
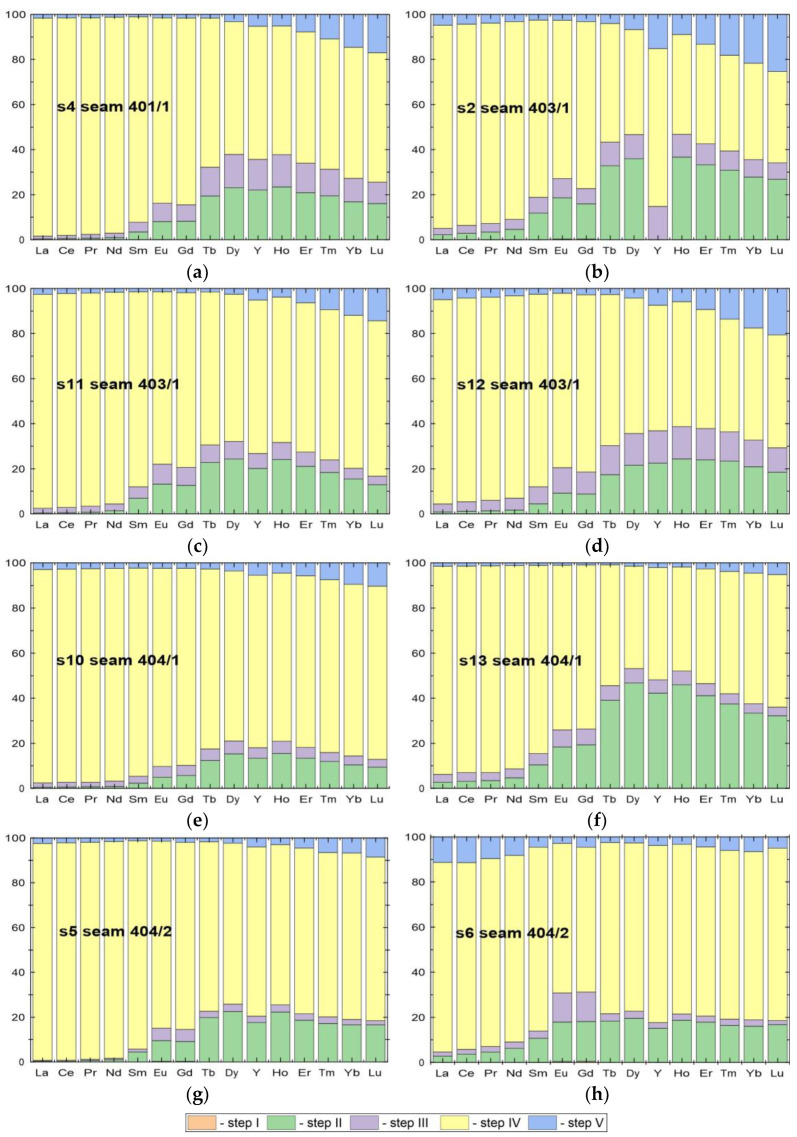
Normalised concentrations of rare earth elements (expressed as a percentage of their total content) in successive steps of sequential chemical leaching of tested samples (**a**–**h**).

The total REY content of the coal samples tested was calculated as the sum of the REY content from the individual sequential leaching steps. It varied between 11.86 and 93.90 ppm (Table 3). The REY concentrations in the samples from seam 404/2 were the lowest of all the samples tested, at 24.85 ppm and 11.86 ppm. Samples from seam 404/1 had the highest ∑REY content, at 92.35 ppm and 93.90 ppm. In the remaining seams (401/1 and 403/1), the ∑REY content ranged from 36.92 to 56.91 ppm. The average ∑REY content in coal of global deposits is 68.52 ppm [4,40]. It was found that the proportion of ∑REY in the samples from seams 401/1, 403/1, and 404/2 was lower, while seam 404/1 had higher ∑REY content than the average for coal of the global deposits. Of the REY analysed, LREY accounted for the highest proportion (>60%) in all samples, while the proportion of HREY did not exceed 5% (Table 3 and Figure 2a).

The ∑REY content of the solutions from steps I–III varied from 1.45 to 18.37 ppm, representing 5.84 to 19.57% of the total REY content of the samples. In these I–III step solutions, MREY tended to account for the largest proportion (>55%). The exception was the s2 sample, in which the proportion of MREY was 41.2%. In solutions from steps IV–V, ∑REY content ranged from 10.42 to 86.18 ppm. The sum of REY concentrations obtained from sequential leaching steps (I–III and IV–V) is consistent with the total REY content of the coal samples, indicating a good mass balance closure.

**Figure 3:**
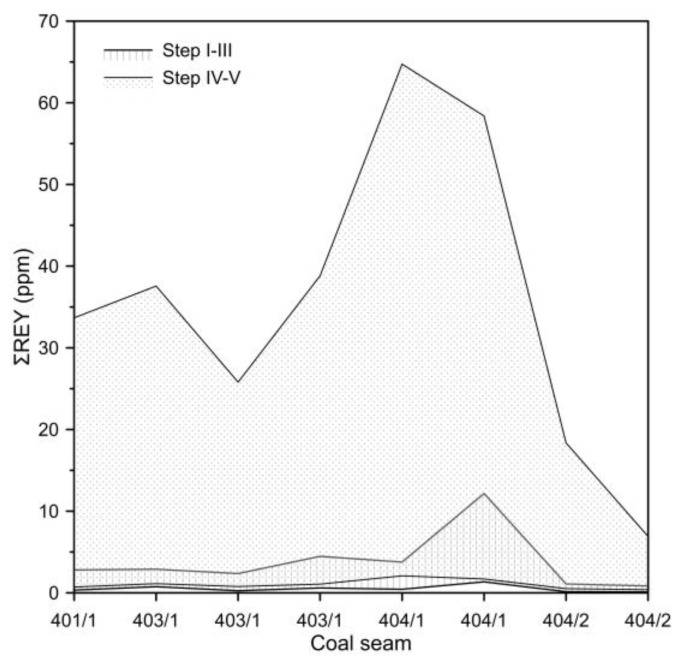
Comparison of REY concentrations in sequential coal leaching solutions in steps I–III and IV–V.

These solutions contained between 80.43% and 94.16% of the total ∑REY in the coal samples. Among the leached REYs, LREYs predominated (66.35–82.11%), while the proportion of HREYs did not exceed 5% (Table 3 and Figure 3).

The proportions of critical (Nd, Eu, Tb, Dy, Y, and Er), uncritical (La, Pr, Sm, and Gd), and excessive (Ce, Ho, Tm, Yb, and Lu) elements were determined in all the sequential coal REY leaching solutions studied. The critical elements content of the coal samples ranged from 5.1 to 39.2 ppm, representing a 32.4 to 43% contribution to the total REY content. The amount of uncritical elements was 3.1–25.1 ppm; their contribution to the total REY content did not exceed 31.1%. In turn, the amount of excessive elements was 3.1–32.2 ppm, representing 31.2–37.0% ∑REY (Table 3 and Figure 1c).

The content of critical elements in the solutions from steps I–III varied from 0.8 to 12.8 ppm, representing 46.9 to 71.3% ∑REY content in these solutions (Table 3). This means that in steps I–III, a proportionally higher amount of critical elements, the proportion of which in coal samples was not higher than 40%, was leached into the solutions. The proportions of uncritical and excessive elements were much lower and did not exceed 31% ∑REY in the solutions from steps I–III.

The content of critical elements in the solutions from steps IV–V varied from 4.3 to 31.1 ppm, representing 29.8 to 41.3% ∑REY content (Table 3). The proportions of uncritical and excessive elements did not exceed 40% of the total REY in these solutions, and the proportions between the three groups of elements were similar to those in the coal samples (Figure 1c).

The results clearly indicate that the highest concentrations of REY in the sequential coal leaching solutions were in those from steps IV–V, where several times higher concentrations were observed compared to steps I–III, particularly in the case of samples from coal seams 403/1 and 404/1 (Figure 3).

### 3.4. REY Anomalies in Coal

**Figure 4:**
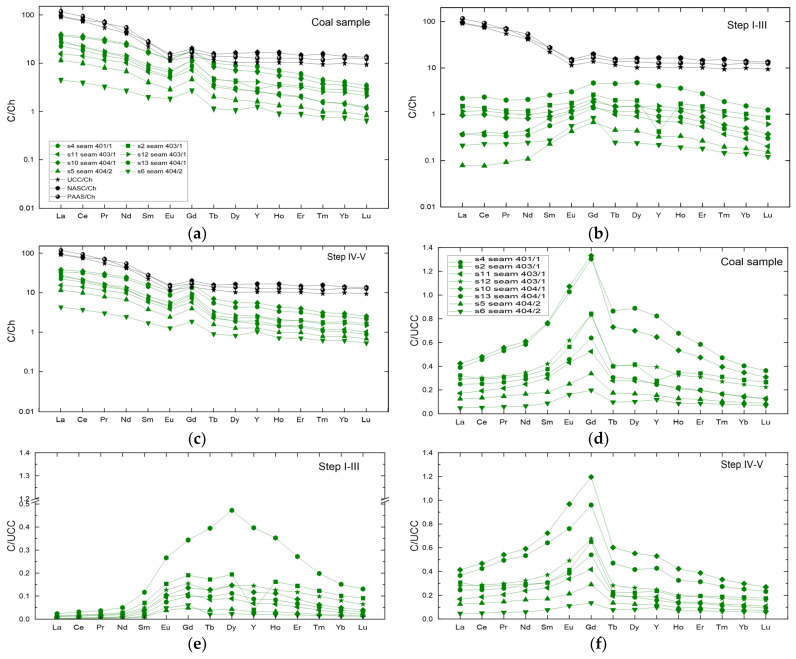
Normalised plots of (**a**–**c**) C/Ch sample/chondrite, (**d**–**f**) C/UCC sample/Upper Continental Crust, REY elements in coal samples and in solutions from their sequential chemical leaching.

REY contents in the coal samples studied were normalised to chondrites. These show a typical enrichment of LREE against HREE and a relatively flat curve for HREY [41] (Figure 4a). Similar trends have been observed in the North American Shale Composite (NASC) and Post-Archean Australian Shale (PAAS), which are standards for comparison purposes when assessing REE content [42]. It can therefore be concluded that the primary source of REE in the studied coal seams in the south-western part of the USCB was clastic material. It cannot be excluded that the observed Eu, Gd, and Y anomalies are partially influenced by diagenetic processes, including redox conditions and fluid interactions, in addition to the primary detrital input.

A small negative anomaly for Eu and a larger positive anomaly for Gd, often occurring in magmatic rocks, including tuffs accompanying coal seams, were observed in all coal samples tested [42,43,44,45]. A slight negative anomaly for Y was also observed in sample s2.

The chondrite-normalised REY contents of the solutions from steps I–III of sequential coal leaching showed a relatively flat curve for LREY (Figure 4b). A positive anomaly covering the entire MREY range was observed in all samples tested. The largest anomaly was observed for Gd, as in the whole coal sample and in NASC and PAAS. A negative anomaly for Y was also evident in the s2 sample.

The curves of REY content normalised to chondrites in solutions from steps IV–V of sequential coal leaching are different compared to steps I–III (Figure 4c). A characteristic enrichment of LREY against HREY and a relatively flat curve for HREY were observed in all curves, and a negative anomaly was observed in the case of Eu and a positive one in the case of Gd. A slight positive anomaly for Y was revealed in sample s6. These curve characteristics virtually correspond to those of the whole coal sample, leading to the conclusion that it was the REY contents of the solutions from steps IV–V that had the greatest effect on the normalised curves of the coal samples (Figure 4a).

### 3.5. Main Types of REY Distribution Patterns

In order to determine the degree of REY enrichment in the samples relative to the content in the Upper Continental Crust (UCC), their proportions in the samples are normalised to those in the UCC [46]. With regard to the distribution of REY content, as compared to UCC, the samples tested can be categorised as follows: LREY-enriched—type L, MREY-enriched—type M, and HREY-enriched—type H. The normalisation curve of each type may have a positive or negative anomaly with different amplitudes relative to Ce, Eu, and Y, as the environmental behaviour of these elements may differ from that of other REY elements. The particular types of REY content distributions are distinguished when (i) La_N_/Lu_N_ > 1—type L, (ii) La_N_/Sm_N_ < 1 and Gd_N_/Lu_N_ > 1—type M, and (iii) La_N_/Lu_N_ < 1—type H. Subtypes and intermediate types can also be distinguished based on the presence of anomalies [4].

The normalisation curves for the coal samples were similar in shape and were of the M-L type. Only in the case of sample s6 was the curve of the M-H type. Over almost the entire range, the curves fall below the reference level. Only for samples s13 and s10 in the Eu–Gd range were the values observed higher than the reference level. The curves show a pronounced bulge in the Nd–Tb range, and in the case of samples s13 and s10, this range extended to Ho. In sample s2, there was a slight negative anomaly for Y. All the normalisation curves analysed were characterised by a maximum at Gd (Figure 4d).

The UCC-normalised REY contents of the solutions from steps I–III of sequential coal leaching showed relatively flat curves for LREY (Figure 4b). A slight positive anomaly was found covering the entire MREY range, with the exception of sample s13. In this sample, a bulge was observed in the Sm–Tm range with a maximum corresponding to Dy. The normalised REY contents for Gd–Ho were nearly twice as high as the values obtained for the other samples. All curves obtained for the REY content of the solutions obtained from steps I–III are M–H-type curves (Figure 4e).

The normalisation curves for the solutions from steps IV–V were of similar shape and were of the M-L type, with the exception of sample s6 representing the M-H type. They showed a clear maximum at Gd and, for samples s2, s6, and s13, a weak positive anomaly at Y (Figure 4f).

### 3.6. Assessment of the Suitability of Coal as a Potential Alternative REY Source

In order to evaluate the tested coal samples as an alternative source of REY, a prospective coefficient (C_outl_) was calculated, as in the case of coal ash, taking into account the proportions of critical and excessive elements, according to the Formula (1):

C_outl_ = (Nd + Eu + Tb + Dy + Er + Y)/(Ce + Ho + Tm + Yb + Lu) (1)

It should be noted that the C_outl_ coefficient is used here primarily as a comparative and classification parameter and does not, by itself, reflect the economic viability of REY recovery without considering absolute element contents and process-related constraints.

**Figure 5:**
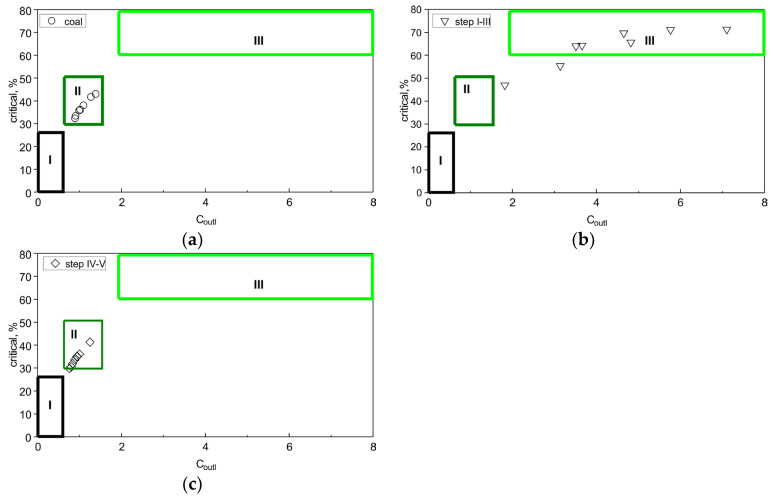
Relationship between the proportion of critical elements and the C_outl_ perspective coefficient for (**a**) coal samples, (**b**) solutions from steps I–III, and (**c**) solutions from steps IV–V against the classification of REY-enriched coal ashes [4]. REY source I—unprospective, II—prospective, III—highly prospective. The C_outl_ coefficient is used here as a comparative classification parameter and should be interpreted together with absolute REY contents.

C_outl_ determination for coal is a factor that should be considered as early as the deposit identification stage, as it allows an indication of the extent to which coal preparation wastes or by-products of coal burning can provide an alternative raw material source for the extraction of critical elements. In addition, in order to determine the potential industrial value, the relation between the percentage of critical elements and C_outl_ was presented (Figure 5a–c).

The C_outl_ value for the coals studied ranged from 0.89 to 1.38, allowing all the samples analysed to be classified as REY prospective raw materials (Figure 5a).

The C_outl_ value of the critical elements in the solutions from steps I–III ranged from 1.82 to 7.10, classifying them as highly prospective raw materials (Figure 5b). However, the proportions of REY in these solutions did not exceed 20% of the total REY content of the coal samples and, at the same time, the absolute REY contents were low (<20 ppm). Therefore, the absolute proportions of REY in the samples are important for the C_outl_ analysis.

The C_outl_ value of the critical elements in the solutions from steps IV–V ranged from 0.76 to 1.25, classifying them as prospective raw materials (Figure 5c). Nevertheless, the proportions of REY in these solutions are much higher than in steps I–III and account for more than 80% of the total REY content in the coal samples, and, at the same time, their absolute contents are several times higher, as mentioned earlier.

## 4. Summary of Test Results

**Figure 6:**
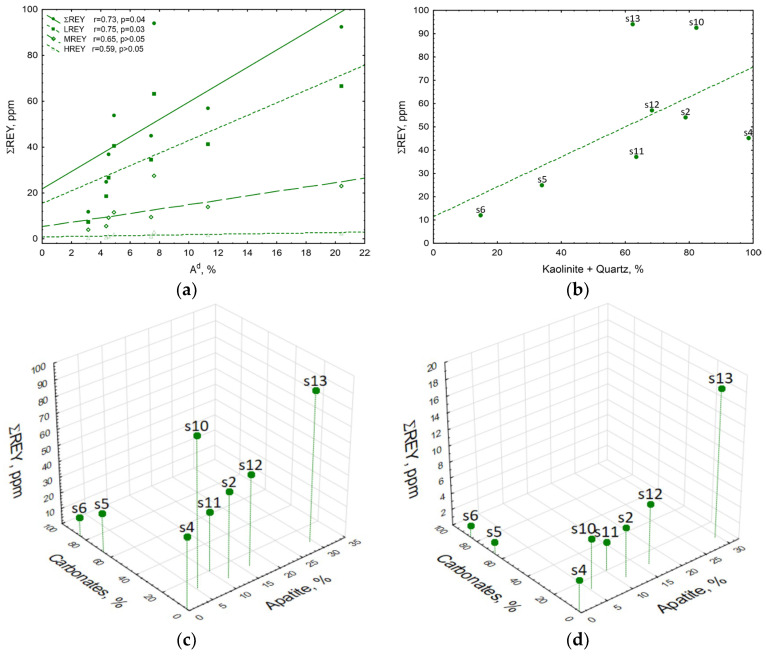
Relationship between (**a**) REY and ash A^d^ content, (**b**) REY and kaolinite and quartz content in coal samples, (**c**) REY content and carbonate and apatite content in coal samples, (**d**) REY content in solutions from steps I–III of sequential chemical leaching of coal and carbonate and apatite content.

The results have revealed that with increasing ash content in the coal samples, the proportion of ∑REY increased, and consequently that of LREY, MREY, and HREY (Figure 6a). The highest increase rate was exhibited for LREY, the proportion of which was also the highest among the REYs analysed. Correlations between A^d^ ash content, ∑REY, and LREY proportions were strong and significant (r = 0.73 and r = 0.75, *p* < 0.05), indicating that ∑REY was related to mineral matter, with LREE having the highest affinity for mineral matter. It was also demonstrated that (i) with increasing kaolinite and quartz content, the proportion of ∑REY increased (Figure 6b); (ii) when apatite was absent in the sample and the greatest amount of carbonates were present (s5, s6), the proportion of ∑REY was lowest (Figure 6c); and (iii) when apatite and small amounts of carbonates were present in the sample (s10, s13), the proportion of ∑REY was highest (Figure 6c).

**Figure 7:**
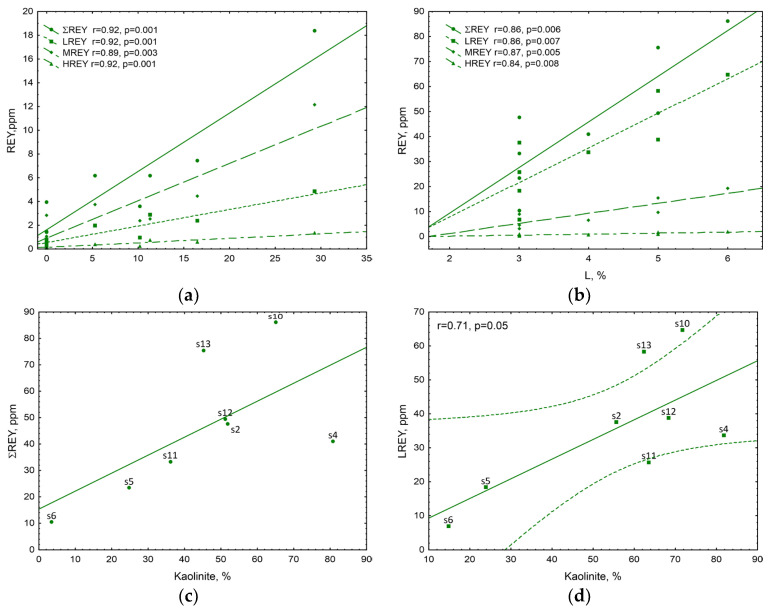
Relationship between REY, LREY, MREY, and HREY content in solutions from sequential chemical leaching (**a**) from steps I–III and apatite, (**b**) from steps IV–V and liptinite; and between (**c**) ∑REE and (**d**) LREY content in solutions from steps IV–V of sequential chemical leaching of coal and kaolinite content in coal samples.

In steps I–III of the sequential chemical leaching of coal samples, mainly carbonates and apatite were transferred into the solutions; however, the results have revealed the following for these solutions: (i) with increasing proportion of apatite in the samples, the proportion of ∑REE increased, (ii) with increasing proportion of carbonates, the proportion of ∑REE decreased (Figure 6d). Noteworthy is the strongly significant positive correlation between ∑REY in solutions and apatite content in coal samples (r = 0.92, *p* < 0.001). Similar trends were observed for the contents of LREY, MREY, and HREY, whereas the highest increase rate was exhibited for LREY, the proportion of which was also the highest among the REYs analysed. All correlations between LREY, MREY, and HREY content and the proportion of apatite were strong and significant (Figure 7a).

In steps IV–V of the sequential chemical leaching of coal samples, mainly silicates and organic matter were transferred into the solutions. The results have shown that in these solutions, with increasing proportion of silicates, the proportion of ∑REE increased (Figure 7c). Noteworthy is the strongly significant positive correlation between LREY in solutions and kaolinite content in coal samples (r = 0.71, *p* < 0.05) (Figure 7d).

REY content was also related to organic matter, as supported by the strongly significant positive correlation between ∑REY and liptinite content in coal samples (r = 0.86, *p* < 0.05). It should be noted that the observed relationship between REY and organic matter is based on correlation analysis and does not allow distinction between direct organometallic associations and indirect associations mediated by mineral phases.

These trends were also observed in the case of LREY, MREY, and HREY; all correlations between LREY, MREY, and HREY content and the proportion of liptinite were strong and significant (Figure 7b).

## 5. Conclusions

In the present study, samples from coal seams from the south-western part of the Upper Silesian Coal Basin (USCB), subjected to five-step sequential chemical leaching, represented medium-rank coal—meta-bituminous B coal. The samples were found to contain varying amounts of siderite, dolomite, calcite, kaolinite, illite, quartz, apatite, and pyrite.

The contents of ∑REY were highest in seam 404/1, with much lower contents in the other seams, while all coal samples featured the highest proportions of LREY (>75%), with HREY proportions below 10%.

The ∑REY contents and the course of the chondrite-normalised REY content curves have revealed that (i) the primary source of REY in the coal seams was clastic material, (ii) the greatest effect on REY content in sequential chemical leaching solutions of coal was in steps IV–V.

In steps I–III, the critical elements were transferred in proportionally greater amounts into the sequential chemical leaching solutions of the coal, while in the case of steps IV–V, the proportions between critical, uncritical, and excessive elements were similar to the proportions in the coal samples.

Based on the value of the prospective coefficient C_outl_, the studied coal samples and solution samples from steps IV–V can be classified as prospective REY sources. However, the research clearly indicates that in the analysis of C_outl_ values, it is necessary to take into account the absolute REY contents of the samples analysed.

∑REE was associated with mineral matter, whereby an increase in apatite and kaolinite content was accompanied by an increase in ∑REE concentration.

## Figures and Tables

**Table 1:** Vitrinite reflectance and maceral group content in the tested coal samples.

Coal Seam	Sample	R_r_, %	s, %	V^mmf^	L^mmf^	I^mmf^	SM	A^d^, %
401/1	S4	1.10	0.04	78	4	18	3	7.42
403/1	S2	1.10	0.04	74	3	23	14	4.92
403/1	S11	1.13	0.04	90	3	7	1	4.53
403/1	S12	1.11	0.04	78	5	17	1	11.29
404/1	S10	1.11	0.04	68	6	26	2	20.40
404/1	S13	1.14	0.04	72	5	23	1	7.64
404/2	S5	1.16	0.04	73	3	24	2	4.39
404/2	S6	1.15	0.05	72	3	25	10	3.16

Explanations: R_r_—mean vitrinite reflectivity; s—standard deviation of mean vitrinite reflectivity; V, L, I—content of vitrinite, liptinite, inertinite, respectively; ^mmf^—state without mineral substance; SM—mineral substance content; A^d^—ash content.

**Table 2:** Maceral group content in the tested coal samples.

Coal Seam	Sample	Siderite	Dolomite	Calcite	Kaolinite	Illite	Apatite	Pyrite	Quartz
		%							
401/1	s4	1.4			81.8	10.4			6.4
403/1	s2	9.2		0.6	55.7		11.3		23.2
403/1	s11	0.8	19.2		63.6		10.2	6.2	
403/1	s12	9.1	2.3		68.4		16.5	3.7	
404/1	s10	12.4			71.7		5.3		10.6
404/1	s13	7.9	0.4		62.4		29.3		
404/2	s5	18.5	47.4		23.9				10.2
404/2	s6	61.7	21.0	2.5	14.8				

**Table 3:** REY contents in the tested coal samples and in solutions obtained from sequential chemical leaching.

	Coal Seam	401/1	403/1	403/1	403/1	404/1	404/1	404/2	404/2
	Sample	s4	s2	s11	s12	s10	s13	s5	s6
	REY	ppm							
Coal	Y	5.38	6.12	5.61	8.65	14.21	18.10	3.41	2.64
	La	7.46	9.69	5.15	8.72	12.70	11.70	3.80	1.48
	Ce	16.07	18.58	12.28	19.36	30.74	29.17	8.62	3.41
	Pr	1.88	2.16	1.51	2.24	3.95	3.77	1.05	0.42
	Nd	7.58	8.34	6.47	8.99	15.88	15.18	4.29	1.70
	Sm	1.49	1.69	1.34	1.89	3.44	3.41	0.81	0.40
	Eu	0.40	0.50	0.38	0.54	0.94	0.90	0.22	0.14
	Gd	2.43	3.20	1.99	3.16	5.06	4.96	1.28	0.75
	Tb	0.20	0.25	0.18	0.26	0.47	0.55	0.11	0.06
	Dy	1.03	1.45	0.97	1.43	2.45	3.11	0.59	0.36
	Ho	0.18	0.28	0.17	0.26	0.43	0.54	0.10	0.07
	Er	0.46	0.78	0.45	0.71	1.09	1.35	0.28	0.20
	Tm	0.05	0.10	0.05	0.09	0.13	0.16	0.03	0.03
	Yb	0.31	0.63	0.32	0.54	0.76	0.89	0.21	0.17
	Lu	0.04	0.08	0.04	0.07	0.10	0.12	0.03	0.02
	∑REY	44.96	53.84	36.92	56.91	92.35	93.90	24.85	11.86
	LREY	34.5	40.5	26.8	41.2	66.7	63.2	18.6	7.4
	MREY	9.4	11.5	9.1	14.0	23.1	27.6	5.6	4.0
	HREY	1.0	1.9	1.0	1.7	2.5	3.0	0.7	0.5
	critical	15.1	17.4	14.0	20.6	35.0	39.2	8.9	5.1
	uncritical	13.3	16.7	10.0	16.0	25.1	23.8	6.9	3.1
	excessive	16.7	19.7	12.9	20.3	32.2	30.9	9.0	3.7
	C_outl_	0.90	0.89	1.09	1.01	1.09	1.27	0.99	1.38
Step I–III	∑REY	3.96	6.19	3.59	7.46	6.18	18.37	1.45	1.45
	LREY	0.81	2.89	0.97	2.39	1.98	4.85	0.22	0.51
	MREY	2.81	2.55	2.36	4.47	3.77	12.17	1.09	0.84
	HREY	0.34	0.75	0.26	0.60	0.43	1.35	0.14	0.10
	critical	2.8	2.9	2.4	4.8	4.0	12.8	1.0	0.8
	uncritical	0.7	1.7	0.7	1.3	1.1	2.8	0.3	0.4
	excessive	0.5	1.6	0.5	1.4	1.1	2.8	0.1	0.3
	C_outl_	5.76	1.82	4.82	3.51	3.65	4.65	7.10	3.14
Step IV–V	∑REY	41.00	47.65	33.33	49.44	86.18	75.53	23.40	10.42
	LREY	33.67	37.56	25.79	38.81	64.74	58.38	18.36	6.91
	MREY	6.63	8.97	6.77	9.57	19.36	15.45	4.52	3.12
	HREY	0.71	1.12	0.77	1.07	2.08	1.70	0.52	0.39
	critical	12.2	14.5	11.7	15.8	31.1	26.4	7.9	4.3
	uncritical	12.6	15.0	9.3	14.7	24.0	21.0	6.7	2.7
	excessive	16.2	18.1	12.4	19.0	31.1	28.1	8.9	3.4
	C_outl_	0.76	0.80	0.94	0.83	1.00	0.94	0.89	1.25

## Data Availability

The original contributions presented in this study are included in the article. Further inquiries can be directed to the corresponding author.
